# The Uncoupling Protein 1 Gene *(UCP1)* Is Disrupted in the Pig Lineage: A Genetic Explanation for Poor Thermoregulation in Piglets

**DOI:** 10.1371/journal.pgen.0020129

**Published:** 2006-08-18

**Authors:** Frida Berg, Ulla Gustafson, Leif Andersson

**Affiliations:** 1 Department of Medical Biochemistry and Microbiology, Uppsala University, Uppsala, Sweden; 2 Department of Animal Breeding and Genetics, Swedish University of Agricultural Sciences, Uppsala, Sweden; Stanford University School of Medicine, United States of America

## Abstract

Piglets appear to lack brown adipose tissue, a specific type of fat that is essential for nonshivering thermogenesis in mammals, and they rely on shivering as the main mechanism for thermoregulation. Here we provide a genetic explanation for the poor thermoregulation in pigs as we demonstrate that the gene for uncoupling protein 1 *(UCP1)* was disrupted in the pig lineage. *UCP1* is exclusively expressed in brown adipose tissue and plays a crucial role for thermogenesis by uncoupling oxidative phosphorylation. We used long-range PCR and genome walking to determine the complete genome sequence of pig *UCP1*. An alignment with human *UCP1* revealed that exons 3 to 5 were eliminated by a deletion in the pig sequence. The presence of this deletion was confirmed in all tested domestic pigs, as well as in European wild boars, Bornean bearded pigs*,* wart hogs, and red river hogs. Three additional disrupting mutations were detected in the remaining exons. Furthermore, the rate of nonsynonymous substitutions was clearly elevated in the pig sequence compared with the corresponding sequences in humans, cattle, and mice, and we used this increased rate to estimate that *UCP1* was disrupted about 20 million years ago.

## Synopsis

Brown adipose tissue (BAT) is unique to mammals. It is rich in mitochondria and generates heat to maintain body temperature during cold stress, referred to as nonshivering thermogenesis. BAT is found in abundance in rodents as well as in newborns of larger mammals, including humans. Uncoupling protein 1 (UCP1) is exclusively expressed in BAT and is localized to the inner membrane of the mitochondria. Its physiological role is to uncouple oxidative phosphorylation so that most of the energy in fat stores is dissipated as heat rather than being converted to ATP.

Piglets are sensitive to cold stress and rely on shivering as the main mechanism for thermoregulation. Furthermore, pigs are the only hoofed mammals that build nests for birth; in modern pig production, heat-producing lamps are used to keep the piglets warm. It is also striking that pigs appear to lack BAT.

Here the authors show that the *UCP1* gene is disrupted in domestic pigs and their wild ancestors. The inactivation of *UCP1* was estimated to have happened about 20 million years ago. The finding provides an explanation for the poor thermoregulation in piglets that may have led to the evolution of the unique maternal behavior in this species.

## Introduction

**Figure 1:**
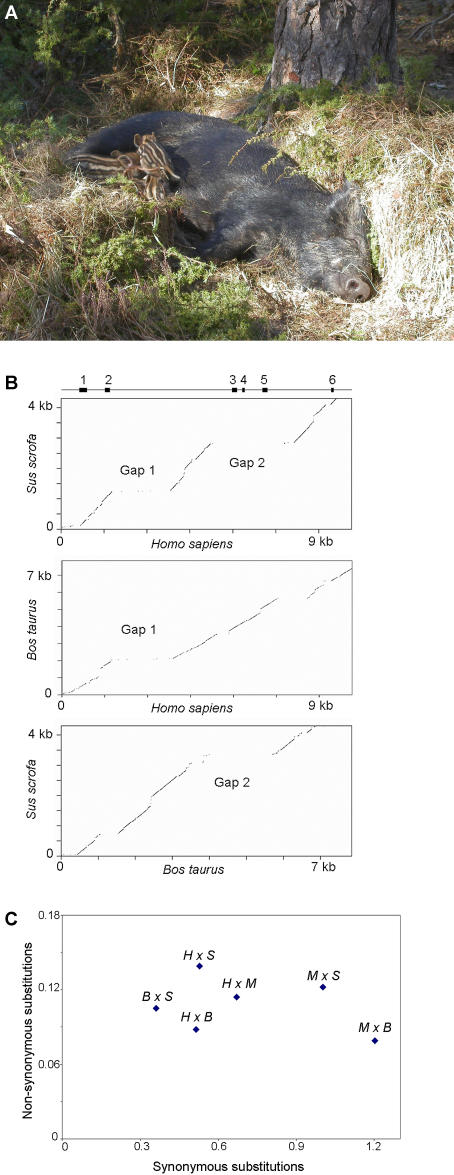
Characterization of the *UCP1* Locus in Pigs (A) Wild boar sow with striped piglets in their nest. (B) Dotpath alignments of genomic *UCP1* sequences from pigs, humans, and cattle. The approximate positions of the six exons in human *UCP1* are indicated. Gap1 and Gap2 indicate two gaps in the alignment between the pig and human sequences. (C) Rate of *nonsynonymous* and *synonymous* substitutions in pairwise comparisons of *UCP1* sequences from humans, mice, cattle, and pigs. The data are based on sequences from exon 1, 2, and 6. B, *Bos taurus;* H, *Homo sapiens;* M, *Mus musculus;* and S, Sus scrofa. (Photo: Anneli Andersson, Linköping University, Sweden)

Pigs are unusual mammals in a number of ways: they are the only ungulates that (1) build nests for birth (Figure 1A), and (2) have large litters, where (3) each young is behaviorally well developed but with a poor thermoregulatory ability [1,2]. It is also striking that piglets appear to lack brown adipose tissue (BAT) [3] and rely on shivering as the main mechanism for thermoregulation [4,5]. BAT is a specific type of fat that is widely expressed in neonatal animals as well as in hibernating rodents [6]. Its physiological role is to generate heat by uncoupling oxidative phosphorylation. Uncoupling protein 1 (UCP1) is exclusively expressed in BAT and plays a crucial role for thermogenesis. UCP1 is located in the inner membrane of the mitochondria, where it catalyzes a regulated proton leakage across the membrane. The established energy is then released as heat [6]. It has been proposed that the acquisition of BAT and UCP1 gave the early mammals an evolutionary advantage by allowing them to be active during periods of nocturnal or hibernal cold and to survive the cold stress at birth [6].

No conclusive evidence for the presence of BAT [7] or for the expression of UCP1 [3,8] has yet been demonstrated in pigs, despite considerable efforts to study this subject. It has therefore been questioned whether pigs express this tissue, and it has been suggested that *UCP1* expression may have been down-regulated during domestication because of strong selection for an efficient energy metabolism. Here we provide an explanation for poor thermoregulation in pigs and the evolution of the unique features in maternal behavior and piglet development. We demonstrate that *UCP1* became disrupted in the pig lineage about 20 million years ago.

## Results

We took advantage of the recent release of a partial pig genome sequence [9] to investigate the porcine *UCP1* locus. The human transcript was blasted against the Sus scrofa trace archive (http://www.ncbi.nlm.nih.gov/Traces/trace.cgi?) by using discontiguous Mega BLAST (National Center for Biotechnology Information, http://www.ncbi.nlm.nih.gov/blast/tracemb.shtml). Two hits, pig trace sequences 812263277 and 782925243, corresponding to *UCP1* exons 2 and 6, were obtained and used for primer design. The complete genome sequence of pig *UCP1* was determined by long-range PCR and genome walking by using genomic DNA from a Large White domestic pig and the porcine bacterial artificial chromosome (BAC) clone PigE-117H8 [10] containing *UCP1*. An alignment with the corresponding human sequence revealed two gaps in the porcine sequence, reducing the total size from 10.1 kilobases in humans to 4.3 kilobases in the pig (Figure 1B). Alignments with cattle *UCP1* showed that Gap1, located in intron 2, represents an insertion in the human lineage or a deletion that occurred before the split of the pig and cattle lineages. Gap2 is unique to the pig and eliminates exons 3 to 5, implying that *UCP1* is disrupted. The presence of this deletion was confirmed by PCR amplification of the deletion breakpoint in pigs representing many different breeds, as well as in European wild boars, Bornean bearded pig *(S. barbatus),* wart hog *(Phacochoerus africanus),* and red river hog *(Potamochoerus porcus)*.

Southern blot analysis using a porcine probe revealed a single restriction fragment consistent with the presence of a single *UCP1* copy in the pig genome (Figure S1). The similarity between the end sequences of the porcine *UCP1* BAC clone and sequences flanking the human *UCP1* gene also supports the interpretation that we have analyzed the true *UCP1* locus and not a defect duplicated version.

The *UCP1* coding sequence in pigs contains three additional disrupting mutations: a two–base pair insertion in exon 1, a 16–base pair deletion in exon 2 (both causing frameshifts), and a nonsense mutation in exon 6 (Figure S2). In contrast, strong purifying selection is observed at the *UCP1* locus in humans, cattle, and mice, as is evident from the 5- to 10-fold higher rate of synonymous (*d*_S_) versus nonsynonymous (*d*_N_) substitutions among these species (Figure 1C). As expected, *d*_S_ was nearly identical when comparing humans with pigs and humans with cattle. In contrast, *d*_N_ was markedly elevated in all pairwise comparisons involving the pig (Figure 1C). For example, human versus pig: *d*_N(H-S)_ = 13.9 ± 2.4%, human versus cattle: *d*_N(B-H)_ = 8.8 ± 1.9%, *Z* = 1.66, *p* < 0.05 in a one-sided test; *Z*=*D*/s(*D*) where *D*=*d*_N(H-S)_ - *d*_N(B-H)_, V(*D*)=V(*d*_N(H-S)_) + V(*d*_N(B-H)_), and s(*D*)=[V(*D*)]^1/2^ [11]. This implies that porcine *UCP1* was inactivated sometime subsequent to the divergence from the cattle lineage and that it has accumulated synonymous and nonsynonymous substitutions at the same rate since then. The inactivation of *UCP1* was dated to have happened about 20 million years ago by using the estimated *d*_N_ rate for the human/cattle comparison and the estimated *d*_S_ rate for the pig/cattle comparison to explain the excess of nonsynonymous substitutions in the pig *UCP1* sequence; the estimated time since the divergence of the human and pig/cattle lineages (94 million years) and the pig and cattle lineages (60 million years) were from Springer et al. [12]. The result is consistent with the observation that *UCP1* is disrupted in several pig species.

## Discussion

We have shown that *UCP1* was inactivated in the pig lineage about 20 million years ago. *UCP1* knockout mice show only a mild phenotype; they are cold sensitive but fully viable [13]. Furthermore, these *UCP1*-null mice develop BAT, which implies that the disruption of porcine *UCP1* cannot by itself explain the absence of BAT. A possible evolutionary scenario is that a pig ancestor lost *UCP1* function and the ability to use BAT for thermoregulation because of no or only weak selection for this mechanism in a warm climate; the wild boar is the only porcine species that has adapted to temperate climates, whereas all other Suidae live in tropical or subtropical environments. The wild boar has then evolved compensatory mechanisms to adapt to a cold environment. Newborn pigs use shivering for thermogenesis [4,5], and the wild boar is the only ungulate that builds a thermoprotective nest for giving birth (Figure 1A). A temperature around +20 °C has been estimated in winter farrowing nests of free-ranging domestic pigs despite outside temperatures down to −20 °C [14]. In modern pig production, heat-lamps are used to facilitate thermocontrol in piglets.

An interesting topic for future research will be to study *UCP1* in more distantly related species to date more precisely the disruption of this gene in the pig lineage. It will be of considerable interest to investigate if the loss of gene expression closely correlates with changes in behavior and litter size among species. Our study does not resolve whether the disruption of *UCP1* was the primary event leading to the loss of BAT or whether it was a secondary event. This question could possibly be resolved by a comparative study of different Suidae species, but it cannot be answered by analyzing sequence data from one species only, because of the poor precision in dating mutation events that happened 20 million years ago. Another important question is whether the loss of *UCP1* and BAT is associated with other genetic changes during the evolution of the pig lineage. For instance, it is possible that the disruption of *UCP1* has led to an upregulation of *UCP2* expression, as observed in the *UCP1* knockout mice [13].

## Materials and Methods

### Animals and BAC resources.

We used genomic DNA representing several different breeds of domestic pigs and samples from European wild boar, wart hog *(P. africanus),* Bornean bearded pig *(S. barbatus),* and red river hog *(P. porcus)*. The pig BAC PigE-117H8 DNA [10] was also used, because its end sequences showed homology to sequences flanking *UCP1* in the human genome (http://www.sanger.ac.uk/cgi-bin/Projects/S_scrofa/WebFPCreport.cgi?mode=wfcreport&name=PigE-117H8).

### PCR analysis.

All primers used for PCR and sequence analysis are listed in Table S1. PCR products were excised from agarose gels and purified using an E.Z.N.A. Gel Extraction Kit (Omega Bio-tek, Doraville, Georgia, United States).

### Long-range PCR.

Forward and reverse primers were located in exon 2 and 6 (primer pair UCP1_A). PCR was performed using Expand Long Template PCR system Mix1 (Roche Diagnostics GmbH, Mannheim, Germany) with the following modifications: 400 μM dNTP, 0.16 M betain, and a final volume of 25 μl. The PCR was run using 1–2 μg of genomic DNA and BAC PigE-117H8 DNA.

### GenomeWalker.

The GenomeWalker universal kit (BD Biosciences, Palo Alto, California, United States) was used to make libraries of Large White pig genomic DNA and BAC PigE-117H8 DNA. PCR was performed using either Expand Long Template PCR system (Roche Diagnostics GmbH) or the AmpliTaq Gold DNA polymerase (Applied Biosystems, Foster City, California, United States).

### *UCP1* exon 1.

The pig trace sequence 848676595 was used to design a forward primer for amplification of the entire exon 1 of *UCP1*. The consensus sequence from the long-range PCR together with the sequences from GenomeWalker were used to design the reverse primer (UCP1_B).

### *UCP1* amplicon spanning Gap2.

A PCR amplicon of about 200 base pairs spanning Gap2 was amplified with the Gap2 primers (Table S1). This amplicon included intron 2 and 5 sequences, showing significant similarity to the corresponding human sequences to ensure that the amplicon spanned the deletion breakpoint.

### Sequencing.

Purified PCR products were sequenced using the forward and reverse PCR primers plus four additional primers for the long-range PCR product: UCP1seq forward and reverse and UCP1seq2 forward and reverse. Sequencing products were generated using the DYEnamic ET dye terminator kit (Amersham Biosciences, Uppsala, Sweden) and separated on a MegaBACE 1000 capillary instrument (Amersham Biosciences). Sequences were aligned using Sequencher 3.1.1 software (Gene Codes, Ann Arbor, Michigan, United States). Some PCR products were cloned into a pCR 2.1-TOPO vector (Invitrogen, Paisley, United Kingdom) to facilitate sequencing.

### Sequence alignments.

*UCP1* sequences were aligned using the dotpath program (http://liv.bmc.uu.se:16080/cgi-bin/emboss/dotpath) (Gary Williams, MRC Rosalind Franklin Centre for Genomics Research, Cambridge, United Kingdom) in EMBOSS explorer [15]. All pairwise alignments of pig, human, and cattle sequences were made**.** *UCP1* exons 1, 2, and 6 from humans**,** mice, cattle, and pigs were aligned using MEGA 2.1 [16] and used to estimate *d*_N_ and *d*_S_. The time since the disruption of the porcine *UCP1* gene was calculated as follows: [(*t*/60) × (*d*_S(B−S)_/2)] + [(2 − *t*/60) × (*d*_N(B−H)_/2 × 60/94)] = *d*_N(B−S)_. The symbols have the following meanings: *t* is the number of years for which *UCP1* has evolved as a pseudogene in the pig lineage; 60 million years is the estimated time since divergence of pig and cattle [12]. *d*_S(B−S)_ is the estimate of *d*_S_ between cattle *(Bos taurus)* and pigs *(S. scrofa)*. We assumed that synonymous substitutions have accumulated at the same rate in the cattle and pig lineage. *d*_N(B−H)_ is the estimate of *d*_N_ between cattle *(B. taurus)* and humans *(Homo sapiens);* 94 million years is the estimated time since divergence of cattle and human [12]. Thus, the expression *d*_N(B−H)_/2 × 60/94 estimates the proportion of nonsynonymous substitutions that have accumulated in the cattle lineage subsequent to the split from the pig lineage 60 million years ago. We assumed that before *UCP1* was disrupted in the pig lineage, it accumulated nonsynonymous substitutions at the same rate as in the cattle lineage. *d*_N(B−S)_ is the estimate of *d*_N_ between cattle and pigs.

## Supporting Information

Figure S1 Southern Blot Analysis Using Genomic DNA Representing Different Pig Breeds Southern blot analysis using genomic DNA representing different pig breeds shows a single *UCP1* fragment. 10 μg of DNA from each individual was digested with HindIII and separated on an 0.7% agarose gel. *UCP1* exon 1, 2, and 6 fragments were used as probes (PCR primers Exon 1, 2, and 6; Table S1); each fragment was labeled separately. No recognition site for HindIII is present in the pig *UCP1* sequence. Lanes 1 and 2, wild boars; lanes 3–11, Large White; lane 12, Duroc; lanes 13 and 14, Hampshire.
(260 KB TIF)

Figure S2 Alignment of Exon 1, 2, and 6 of Human, Mouse, Cattle, and Pig *UCP1,* and the Corresponding Amino Acid Sequences Identities to the master sequence (human) are marked with dots, and missing data/gaps are indicated by dashes. (A) The alignment reveals a two–base pair insertion in exon 1 and a 16–base pair deletion in exon 6 of the pig sequence, indicated by stars. Arrows point out the first nucleotide in exon 2 and 6. (B) The insertion differences were ignored when the pig sequence was translated. The translated sequence reveals a premature stop codon in the pig sequence.
(76 KB TIF)

Table S1 A List of Primers Used to Amplify and Sequence the Porcine *UCP1* Gene (25 KB DOC)

### Accession Numbers

The pig sequence has been deposited in GenBank (http://www.ncbi.nlm.nih.gov/Genbank) under accession number DQ372918.

The Ensembl (http://www.ensembl.org/index.html) accession numbers for the genes and transcripts in this paper are cattle gene (ENSBTAG00000004647), cattle transcript (ENSBTAT00000006097), human gene (ENSG00000109424), human transcript (ENST00000262999), and mouse transcript (ENSMUST00000034146).

## Abbreviations

BAC: bacterial artificial chromosome
BAT: brown adipose tissue
*d*_N_: number of nonsynonymous substitutions per nonsynonymous site
*d*_S_: number of synonymous substitutions per synonymous site
UCP1: uncoupling protein 1
