# Supplementary material for: The Uncoupling Protein 1 Gene (UCP1) Is Disrupted in the Pig Lineage: A Genetic Explanation for Poor Thermoregulation in Piglets
Source: PLoS Genet. 2006 Aug 18;2(8):e129. doi: 10.1371/journal.pgen.0020129 (PMC1550502; doi:10.1371/journal.pgen.0020129)
Supplement: Supplementary file 1 [file pgen.0020129.st001.doc]

**Supplementary Figure 1**. Southern blot analysis using genomic DNA representing different pig breeds shows a single *UCP1* fragment. Ten µg DNA from each individual were digested with *Hind*III and separated on an 0.7% agarose gel. *UCP1* exon 1, 2, and 6 fragments were used as probes (PCR primers Exon1, 2 and 6, Supplementary Table 1); each fragment was labeled separately. No recognition site for *Hind*III is present in the pig *UCP1* sequence. Lane 1 and 2; wild boars, lane 3-11; Large White, lane 12; Duroc, lane 13 and 14; Hampshire.

**Supplementary Figure 2**. Alignment of exon 1, 2 and 6 of human, mouse, cattle and pig *UCP1,* as well as the corresponding amino acid sequences. Identities to the master sequence (human) are marked with dots and missing data/gaps are indicated by dashes. a) The alignment reveals a two bp insertion in exon 1 and a 16 bp deletion in exon 6 of the pig sequence, indicated by stars. Arrows point out the first nucleotide in exon 2 and 6. b) The insertion differences were ignored when the pig sequence was translated. The translated sequence reveals a premature stop codon in the pig sequence.
